# *Lean* and leadership practices: development of an initial realist program theory

**DOI:** 10.1186/s12913-015-1030-x

**Published:** 2015-09-07

**Authors:** Donna Goodridge, Gill Westhorp, Thomas Rotter, Roy Dobson, Brenna Bath

**Affiliations:** Department of Medicine, College of Medicine, University of Saskatchewan, Room 543 Ellis Hall, 108 Hospital Drive, Saskatoon, SK S7N 0W8 Canada; Community Matters, Inc., Professorial Fellow, Charles Darwin University, P.O. Box 443, Mt Torrens, SA 5244 Australia; Chair in Health Quality Improvement, College of Pharmacy and Nutrition, University of Saskatchewan, Health Sciences Building, 104 Clinic Place, Saskatoon, SK S7N 2Z4 Canada; Social and Administrative Pharmacy, College of Pharmacy and Nutrition, University of Saskatchewan, Room E3112, Health Sciences Building, 104 Clinic Place, Saskatoon, SK Canada; Department of Physical Therapy, College of Medicine, University of Saskatchewan, Room 215, 1121 College Drive, Saskatoon, SK S7N 0W3 Canada

## Abstract

**Background:**

Lean as a management system has been increasingly adopted in health care settings in an effort to enhance quality, capacity and safety, while simultaneously containing or reducing costs. The Ministry of Health in the province of Saskatchewan, Canada has made a multi-million dollar investment in Lean initiatives to create “better health, better value, better care, and better teams”, affording a unique opportunity to advance our understanding of the way in which Lean philosophy, principles and tools work in health care.

**Methods:**

In order to address the questions, “What changes in leadership practices are associated with the implementation of Lean?” and “When leadership practices change, how do the changed practices contribute to subsequent outcomes?”, we used a qualitative, multi-stage approach to work towards developing an initial realist program theory. We describe the implications of realist assumptions for evaluation of this Lean initiative. Formal theories including Normalization Process Theory, Theories of Double Loop and Organization Leaning and the Theory of Cognitive Dissonance help understand this initial rough program theory. Data collection included: key informant consultation; a stakeholder workshop; documentary review; 26 audiotaped and transcribed interviews with health region personnel; and team discussions.

**Results:**

A set of seven initial hypotheses regarding the manner in which Lean changes leadership practices were developed from our data. We hypothesized that Lean, as implemented in this particular setting, changes leadership practices in the following ways. Lean: a) aligns the aims and objectives of health regions; b) authorizes attention and resources to quality improvement and change management c) provides an integrated set of tools for particular tasks; d) changes leaders’ attitudes or beliefs about appropriate leadership and management styles and behaviors; e) demands increased levels of expertise, accountability and commitment from leaders; f) measures and uses data effectively to identify actual and relevant local problems and the root causes of those problems; and g) creates or supports a ‘learning organization’ culture.

**Conclusions:**

This study has generated initial hypotheses and realist program theory that can form the basis for future evaluation of Lean initiatives. Developing leadership capacity and culture is theorized to be a necessary precursor to other systemic and observable changes arising from Lean initiatives.

## Background

Contemporary health care systems are under mounting pressure to improve patient outcomes while simultaneously achieving greater efficiencies - “to do more with less” [1]. Industrial improvement solutions, such as *Lean*, offer the seductive promise of enhancing quality, capacity and safety in the health care environment, while containing or reducing costs [2]. A recent Conference Board of Canada survey of Canadian health regions [3] reported that, while 73 % indicated that *Lean* was a component of their organizational strategy, few have embraced *Lean* as an overarching strategy and management system designed to transform organizational culture and performance [3].

The Conference Board of Canada survey [3] noted that three provinces (Manitoba, New Brunswick and Saskatchewan) mandate all health regions to participate in *Lean*, while only Nunavut reported not using *Lean* in health care [3]. Secondary and tertiary care settings, particularly those areas where high volume and rapid processing needs exists (e.g. laboratories and emergency departments), accounted for 72 % of all *Lean* activities, while primary and community settings did not make as much use of *Lean* [3].

Saskatchewan was noted to be the only province committed to a consistent and comprehensive implementation of *Lean* across the entire health system [3]. In what has been billed as “the largest *Lean* transformation in the world” [4], the Ministry of Health in the province of Saskatchewan, Canada (population 1.12 million – Bureau of Statistics, 2014), has made a province-wide, multi-million dollar investment in *Lean* initiatives, with aim of “thinking and acting as one” to create “better health, better value, better care, and better teams” [5]. These goals will be achieved through “developing infrastructure to support and coordinate continuous quality improvement” as well as “building capability among leaders and the entire healthcare workforce to do daily continuous improvement” [6]. An external consulting company with previous experience in implementing *Lean* in health care settings was engaged by the province.

This large scale transformation effort has afforded a unique opportunity to advance our understanding of the way in which *Lean* philosophy, principles and tools work in health care, both from a practical as well as from a theoretical perspective. An initial description of *Lean* implementation in Saskatchewan has been provided in a previous publication [7]. The following paper reports on a realist approach to develop initial program theory about the role of leadership in the Saskatchewan model of *Lean.* During the baseline phase, theory development focused on developing an ‘initial rough program theory’, i.e. an understanding of how the program is intended to work. Future stages will concentrate on developing *realist* program theory, i.e. an understanding of the circumstance in which, and mechanisms by which, the program works (when it does) or does not work (when it doesn’t).

The present paper deals with two questions. Firstly, what changes in leadership practices are associated with the implementation of *Lean*? Secondly, when leadership practices change, how do those changed practices contribute to subsequent outcomes? We propose some tentative hypotheses about circumstances relating to leadership for investigation in future stages of the work.

### Lean

Originally derived from the Toyota car company production line system the *Lean* paradigm builds on the significant history of work relating to structured process improvement. Adaptations of the Toyota Production System (TPS), which originated in Toyota factories after the end of World War II, are often termed *Lean* initiatives because of the focus on “devising nimble tasks, processes and enterprises that maximize value and minimize waste in all of its forms” [8]. Emblematic *Lean* practices such as ‘just-in-time’ (producing only what is needed by the next process in a continuous flow) and ‘kanban’ (pull control of operations) characterize much of Lean implementation in industries outside of health care.

Health care systems have increasingly recognized the potential value of the results-oriented *Lean* paradigm to make a valuable contribution to wicked and long-standing problems such as inefficiencies, lack of consumer-centredness and spiraling costs that continue to plague the industry. The Lean paradigm uses approaches that are relatively novel in health care, such as management of minimal inventories, minimal buffers of work-in-process and minimal latitude in planning [9]. Lean has appeared in diverse incarnations in health care. The Institute for Healthcare Improvement has produced a White Paper comparing *Lean* and Quality Improvement [8], to which readers are referred for a more in-depth discussion of *Lean* in health care. We are also conducting a systematic review on Lean management in health care in the overarching evaluation of Lean implementation in Saskatchewan [10]. We will publish the results of our systematic review on the definition, implementation strategies and effects reported in the literature.

However, application of a quality improvement system that has been proven to work in a high volume/low variety industrial system such as manufacturing to the low volume/high variety/high variation in demand environment of health care has been met with some reasonable skepticism, particularly given that strategic performance indicators may be different for car plants and health care settings [9]. Given the complexity of hospital environments and the diversity of the operations required to meet their needs, certain Lean techniques may be more appropriate for some departments than others. For example, Lean techniques such as ‘kanban’ may lend them more easily to managing equipment in highly structured laboratory settings than to managing equipment in the typically chaotic environment of an emergency department.

The Saskatchewan model of *Lean* [11] represents a patient-centred, multi-faceted approach to organizing and delivering healthcare. *Lean* is a set of operating philosophies, tools and management activities that help create maximum value for patients by reducing the sources of waste in a process [10, 12]. *Lean* partners a philosophy of empowerment with a highly structured set of methods in order to deliver higher quality services at lower cost [3]. *Lean* tools and techniques aim to reduce waste and enhance productivity through reconfiguring organizational processes [13]. Customers’ needs and desires drive continuous improvement in the quality of services and products [3]. The “true experts” in the process of health care (e.g. patients and families, healthcare providers and support staff) are engaged in a continuous learning cycle with the support and coaching of their leaders [14]. While the Saskatchewan model of Lean incorporates emblematic tool and techniques such as ‘kanban’ and ‘Jidoka’ (stopping work when a problem first occurs), our focus in this paper is on the social and collective aspects of the Lean model.

Value is a key element of the *Lean* process and has three dimensions within health care: 1. Clinical value (achieving the best possible patient outcomes); 2. Operational value (efficiency, accessibility and continuity of care); and 3. Experiential value (experiences of patients and providers and reflected in patient satisfaction and employee work life) [15]. *Lean* processes work as diagnostic tools to capture the sources of waste and identify areas of possible improvement.

Spear and Bowen [16] distilled the tacit knowledge that underlies the Toyota Production System into four basic rules: 1. All work is highly specified in its content, sequence, timing and outcome; 2. Each worker knows who provides what to him and when; 3. Every product and service flows along a simple, specified path; and 4. Any improvement must be made using the scientific method, under a teacher’s guidance, and at the lowest possible organizational level.

*Lean* philosophy is premised on five key principles as depicted in Table 1.

**Table 1:** Five Key Principles of *Lean* [34]

Identify customers and specify value	This principle acknowledges that only a small proportion of the time and effort in any organization adds value for the customer. Value for a specific product or service must be clearly defined *from the customer’s perspective.* Non-value activities are considered “waste” and targeted for removal.
Identify and map the value stream	The value stream represents the whole set of activities across all parts of an organization involved in jointly delivering the product or service. Once there is understanding of what the customer wants, the organization moves on to identifying how the delivery is occurring.
Create flow by eliminating waste	Eliminating waste results in the product or service seamlessly “flowing” to the customer without detours, interruption or waiting.
Respond to customer “pull”	The process is created based upon the organization’s understanding of customer demand, producing what is wanted when it is wanted
Pursue perfection	As radical reorganization occurs, gains becoming increasingly significant when all the steps link together. Perfection is the theoretical end-point, occurring when every asset and action adds value for the customer

*Lean* is one of many different industrial management systems that have been adopted to improve healthcare quality over the past twenty years. Mackenzie and colleagues [3] suggest that *Lean* differs from conventional process improvement approaches in several ways. Conventional approaches focus on increasing the productivity of value-added processes, requiring employees to work both harder and faster. The benefits are typically confined to a specific process with little impact occurring at the department or system level. *Lean* specifically targets non-value-added processes for elimination across the entire value stream and is applied across the entire patient journey in an organization [3].

Despite *Lean*’s focus on measurement and evidence, rigorous evaluations of the operational impacts at a system level and consideration of organizational culture are few [3, 17]. Puterman et al. (2013) describe a framework for evaluation of *Lean* interventions that include key components, such as efficiency gains; quality and safety improvements; staff engagement enhancements; and financial and resource inputs [17]. The existing evaluation research to date on *Lean* implementation tends to focus on specific clinical outcomes. For example, a recent comparison of Emergency Department wait times demonstrated no difference between 36 Ontario hospitals that had implemented *Lean* and 63 matched control sites without the *Lean* program [18]. Complex interventions such as Lean, which is a deliberately initiated attempt to engineer patterns of collective action in health care, are particularly difficult to evaluate because the components of the intervention may act independently or interdependently, and the relationships between them become challenging to parse out [19]. In particular, the social relations and processes must be evaluated alongside outcomes and effectiveness [19]. Until comprehensive, theory-driven evaluations of *Lean* implementation in health care become more commonplace, our understanding of how and why *Lean* may work remains incomplete.

Failures of *Lean* implementation in the business world are common. A survey conducted by Industry Week in 2007 found that only 2% of companies with a *Lean* program fully achieved their objectives and less than one quarter reported achieving significant results [20]. These failures have been attributed to a range of factors, including: a lack of commitment by senior management; unwillingness by senior management to accept the cultural change required for *Lean* to be a success [20, 21]; or a fundamental misunderstanding of the Toyota Production System in practice [22]. Mann [23] notes that *Lean* is a “high-touch, high-maintenance enterprise”, requiring ongoing vigilance by leaders to ensure regression to customary ways of conducting business does not occur. Given the stated importance of leadership to *Lean* implementation, we turn to a specific discussion of *Lean* and leadership.

### Lean and leadership

Strong leadership is recognized to be critical to successful implementation of *Lean*. While *Lean* tools are often the most visible component of implementation, Mann [23] suggests that “80 % of the effort [in *Lean* implementation] is expended on changing leaders’ practices and behaviors, and ultimately their mindset”. Strategic leadership activities that facilitate successful implementation of *Lean* include: developing governance arrangements that cross divisional boundaries; supporting a comprehensive, long-range vision of the organization’s value-producing processes; and holding people accountable for meeting *Lean* commitments [23].

In the previously cited Conference Board of Canada (2014b) survey, senior leadership involvement was the most highly rated success factor by respondents. In 41 % of cases, senior leaders sponsored *Lean* activities by motivating others, establishing goals and removing barriers, while 30 % supported *Lean* by delegating duties and being involved in management activities as appropriate. Others championed *Lean* by being actively involved and “modelling the way” [3]. Mackenzie & Hall [3] note that leadership commitment and creation of a vision involving *Lean* are pivotal to “stakeholders’ understanding of the benefits of *Lean* for themselves or for the organization as a whole”.

Different levels of the leadership hierarchy assume complementary, yet overlapping, roles in the implementation of *Lean* [23]. For example, the primary contribution of the strategic leadership provided by senior management is likely to be governance, steering and oversight, while the primary contribution of front line managers and supervisors focuses on tactical leadership, including teaching and practicing root cause problem-solving [21]. In order to achieve the cultural shift needed to ensure success in *Lean* initiatives, leaders at all levels of the organization must learn to reinforce behaviors that may not have been highly valued in the past [21]. While patients and families have often complained of “having no voice” in health care, *Lean* highlights the value of listening to and acting upon consumer insights to improve quality. The acts of listening and responding to patients and families become more valued and new strategies to promote these behaviors must be incorporated into leaders’ repertoires. Another example of changed priorities in behaviors is leadership visibility and engagement. Given the competing and often urgent demands faced by leaders, regular engagement with front-line workers is typically accorded a low priority in many workplaces. Lean changes the priorities of leaders by emphasizing the importance of this type of engagement. “Gemba walks”, in which leaders go to the “shop floor” to examine process and speak with workers, reinforce *Lean* practices and engages the leader in experiential learning about implementation [21].

Standardized work is required of leaders in *Lean* implementation, just as it is of workers, and has been described as a “culture change inside a culture change “. Standardized work for leaders involves: 1) auditing and verification of direct reports; b) defining outcome metrics and c) leadership tasks, including scheduled, unscheduled but predictable and unscheduled and unpredictable tasks [24]. Scheduled tasks are those that must be completed and occur regularly, e.g. completing payroll. An example of an unscheduled but predictable task would occur when emergency department overcrowding necessitates consideration of which patients could be discharged home. Unscheduled and unpredictable tasks may occur in relation to an outbreak of influenza among patient and staff. Standardized work takes into account these three scenarios and provides documentation on how to address these situations.

Within Saskatchewan, the commitment has been made by government to train 880 “*Lean* leaders” over a three year period. *Lean* leader training represents an enormous commitment of resources by the individual trainee, the health region and the government, which funds the health regions. Leader training involves a series of courses, including Value Stream Mapping, a series of 24 *Lean* training modules, a “Module Deep Dive” to deepen understanding, a “Module Marathon” in which participants must present their learnings, participation in a Rapid Improvement Workshop (a focused improvement event) as well as participation in a study tour of organizations in North America for those further ahead on their *Lean* leader training journey.

In this paper, we focus specifically on how *Lean* may change leadership practices, with a view to contributing to the evidence base on *Lean*. The first stage of a realist approach is developing ‘program theory’ about the specific issue under investigation, and it is this theory that is our particular focus here.

### The realist approach

Given the highly complex nature of the *Lean* intervention, the team chose to work towards a realist approach for evaluation following initial exploratory work (described below). The term “realist” is drawn from Pawson and Tilley’s seminal work, Realistic Evaluation [25]. As the name suggests, this is an approach grounded in realism, a school of philosophy which asserts that both the material and social worlds are ‘real’ and can have real effects; and that it is possible to work towards a closer understanding of what causes change. *Mechanisms* describe how the resources embedded within a program influence the reasoning and the behavior of program participants [26].

A realist approach assumes that programs are ‘theories incarnate’. That is, whenever a program is implemented, it is testing a theory about what ‘’might cause change’, even though that theory may not be explicit. One of the tasks of a realist evaluation is therefore to make the theories within a program explicit, by developing clear hypotheses about how, for whom, and in which contexts the intervention might ‘work’. The evaluation then tests those hypotheses. Program theories are related back to formal theories to assist in abstraction and to access previous research relevant to the program theory. In this paper, we draw on three formal theories: May’s Normalization Process Theory; Argyris’ Theories of Double Loop and Organizational Learning; and Festinger’s Theory of Cognitive Dissonance to help understand our initial rough program theory [19, 27, 28].

The realist approach includes a number of key assumptions and we outline their implications for our *Lean* evaluation in Table 2.

**Table 2:** Implications of Realist Assumptions for Evaluation of *Lean* Implementation in Saskatchewan

Assumption	Implication for Evaluation
*Social programs are an attempt to create some kind of change.*	The implementation of *Lean* aims to improve the quality and efficiency of the Saskatchewan health system.
*Programs ‘work’ by enabling participants to make different choices, understanding that choice making is constrained by participants’ previous experiences, beliefs and attitudes, opportunities and access to resources.*	Different types of decision makers will influence *Lean* outcomes, from those in senior positions at the central level through unit administrators, team leaders, practitioners and patients. A wide variety of factors will affect the decisions that they make.
*The context in which a program operates will make a difference to the outcomes the program achieves. The context features aspects such as social, economic and political structures, organizational context, program participants, program staffing, geographical and historical context, and so on.*	Context can influence program mechanisms and outcomes in many different ways.
	Organizational culture varies across regions and across types of health units. Professions and occupations have different norms. Culture, gender and socialization shape patterns of decision-making. Organization priorities may influence the ways in which, or the extent to which, particular *Lean* approaches are implemented, who it targets, who it reaches and so on.
	Access to resources to implement decisions, and opportunities to implement decisions, can also influence reasoning itself, as well as whether or not desired choices can be put into action.
*Because there is always an interaction between context and mechanism, that interaction is what determines the program’s impacts or outcomes: Context + Mechanism = Outcome.*	Testing the ‘CMO hypotheses’ requires data about each element of the hypothesis; the context, mechanism, and outcome. It also requires analytic techniques that can identify the relationships between them.
*Because programs work differently in different contexts and through different change mechanisms, programs cannot simply be replicated from one context to another and be expected to automatically achieve the same outcomes.*	At the macro level, it cannot be assumed that *Lean* will work in the same ways, or to the same extents, in different kinds of health units or different regions.
	At the micro level, it cannot be assumed that a solution generated in one setting will necessarily work in another setting.
*Good understandings about ‘what works for whom, in what contexts, and how’ are, however, portable.*	A realist evaluation should generate a deep understanding of how and why *Lean*, or *Lean* tools, works well in some contexts and less well in others. This may assist policy makers and administrators to adapt to their own contexts, and thus to improve outcomes. This is entirely consistent with the ‘local solutions’ principle of *Lean* itself.

## Methods

### Study design and methods

To address the questions related to leadership and *Lean*, we used a qualitative approach. A primary goal of qualitative research is to produce knowledge that is transferable from one context to another. From a realist perspective, this goal is approached by developing an initial rough theory about how an intervention is expected to work – that is, by identifying the underlying mechanisms that are expected to cause (or more precisely contribute to) change. Data is then analyzed to understand ‘what it is about the context’ that affects whether and how interventions work in the expected ways, what outcomes they generate in different contexts, and why.

Theory development for realist evaluation can draw on multiple sources. The exploratory work that was conducted, and later used as sources of data to develop our initial theory regarding how *Lean* implementation may change leadership, included:

- An initial stakeholder workshop conducted in June 2013, attended by Health Quality Council staff, regional health staff, clinicians, patients and members of the research team;
- Review of *Lean* documentation, and in particular the training resources available on the Ministry of Health website;
- An initial set of interviews with personnel in health regions, described further below;
- Discussions amongst research team members.

#### Preliminary key informant and stakeholder consultation

Nine interviews were conducted by two members of the research team with key informants to solicit expert opinion on the direction the *Lean* evaluation could most fruitfully take. The research team also facilitated an interactive stakeholder consultation workshop in June, 2013. Forty-nine stakeholders attended, including knowledge users and decision makers, such as clinicians, patients, families and managers, representing twelve health regions across the province. The over-arching question explored during this meeting was ‘For whom, how and under what circumstances is the *Lean* Management System effective in improving care, health, value and teamwork?’

Participants worked in assigned small groups, facilitated by research team members who kept notes on the discussion. The groups described the behavior changes, reasoning, and outcomes of work being conducted in each of the following *Lean* approaches: Kaizen (term for the process of continuous improvement) Basics; Rapid Process Improvement Workshops; Leadership Training; Value Stream Mapping; Kaizen Promotion Offices; Visual Daily Management; and Hoshin Kanri (a province-wide planning process running alongside *Lean* implementation). The data related to each of these areas were transcribed and collated to inform the questions asked in the subsequent round of interviews, and to inform the first phase of theory development.

#### Lean documentation

The training resources available on the Saskatchewan Health Quality Council website [29] were reviewed in detail as part of a documentary analysis. The review sought to extract both ‘what is supposed to be done’ (that is, the theory of action) and ‘how it is supposed to be done and why it is supposed to be done that way’ (the ‘why’ usually provides clues about the intended theory of change). Particular attention was paid to changed roles and expectations of leaders and staff because these relate to the two overarching mechanisms being investigated in this evaluation (changed leadership practices and staff empowerment).

#### Interviews

Ethical approval to conduct the interviews was obtained from the University of Saskatchewan (Beh# 13-294). Written consent was obtained from participants and transcribed interviews were provided to participants for editing and approval of transcript release.

Based upon the data gathered through key informant interviews, the stakeholder consultation and the review of *Lean* documentation, an initial interview guide was designed by the team. Because the research team, at that time, needed an overview of the changes perceived to be associated with *Lean*, we did not structure the questions specifically to conform to the realist focus on contexts, mechanisms and outcomes. Rather, the interview guide sought to gather evidence about perceptions regarding changes to respondents’ work in the health system or experiences in the health system over the past two years, the respondents’ experience in *Lean* and what they most valued about it; changes that they attributed to *Lean* in relation to patient care and outcomes, relationships between patients and staff, staff and co-workers, and staff and leaders; adaptations of tools for the local context; the pace of change; concerns about *Lean* and adequacy of resources.

The primary source of evidence described in this paper was derived from these face-to-face interviews which contributed to, or provided preliminary evidence for, aspects of the program theory in relation to leadership change. Given the early stage of the research (this was a baseline study, *Lean* had only recently been introduced in most regions and the interviews were conducted before the focus on leadership had been selected), we anticipated greater depth of information about the first stage of change – how *Lean* changes leadership practice. Because a realist approach also expects that things will work differently in different contexts, we also sought initial evidence about the contexts in which changes appeared to be beginning and those in which less progress was evident.

Audiotaped interviews were conducted in a private space by the trained research assistant using a semi-structured interview guide. The research assistant had extensive previous experience in interviewing, and received additional training in the fundamentals of realist approaches. Ethical approval was obtained from the University of Saskatchewan Behavioral Ethics Board and operational approval granted from regional authorities as per their usual protocol.

Verbatim transcripts of the interviews were obtained and reviewed in detail by the two lead members (DG, GW) of the qualitative team. A coding framework was developed to address the analytic tasks of explaining outcomes by identifying mechanisms which create the change and identifying the contexts that influence: if mechanisms “fire”; which mechanisms “fire” and for whom mechanisms “fire”. Coding was conducted independently by each researcher followed by extensive discussion between the researchers. Summaries were presented to three other members of the qualitative research team, who had full access to the verbatim transcripts, for feedback and further discussion prior to Context-Mechanism-Outcome configurations (CMOCs) being developed by the primary qualitative researchers. The draft CMOCs were presented back to the entire research team and revised based on the discussion. Given the large volume of data arising from these interviews, this paper focuses specifically on: a) how *Lean* changes leadership practices and; b) when leadership practices do change, how do those changed practices contribute to later outcomes?

#### Sample and setting

Kaizen Promotion Offices had been established in five of the 13 health regions across Saskatchewan to build capacity for continuous improvement by promoting *Lean* principles and tools. Kaizen Promotion Office (KPO) Directors or their equivalent, from four health regions in Saskatchewan (one large urban, two small urban, and one northern) were asked to identify patients, front-line staff and leaders who had participated in a *Lean* activity (Rapid Process Improvement Workshops - RPIW) within the previous six months and who might be amenable to discussing their experiences with *Lean* for this project.

## Results and discussion

Fifty-two potential interview participants were contacted with a participation rate of 51.9 %. A minimum of three attempts were made to contact each individual and an alternate list of potential participants was requested when there was insufficient participation from the original list.

A total of 26 face-to-face and one telephone interview were conducted. These were comprised of: six participants from the large urban region; seven from one small urban region and eight from the other small urban region; and six from the northern region. The roles of respondents were varied and included one CEO, five directors, four managers, four allied health clinicians, three registered nurses, two clerical/administrative staff, two physicians and six patients.

**Fig. 1:**
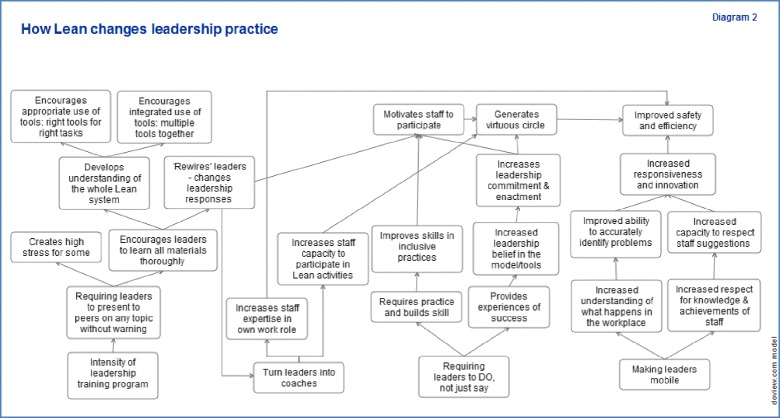
*Lean* and Leadership Practice

Use of the realist coding matrix was helpful in directing attention to emerging CMOCs, although the nature of the questions meant that that much of the interview data related fairly specifically to the interim outcomes in which *Lean* implementation was occurring rather than to mechanisms and outcomes. However, triangulating interview data with our other sources of data (key informant interviews, stakeholder consultation and *Lean* program documentation) allowed us to develop a detailed theory diagram (Fig. 1) representing program theory about how *Lean* might work to change leadership practices. A series of initial hypotheses (Table 3) specifying how *Lean* might change leadership practices were then distilled from these theory diagrams and will be used for future testing in future longitudinal research.

**Table 3:** Initial Hypotheses Regarding *Lean* and Leadership

*Lean, as implemented within Saskatchewan, changes leadership practices* by:
1. Aligning the aims and objectives of health regions across the province;
2. Authorizing attention and resources to quality improvement and change management;
3. Providing an integrated set of tools for particular tasks;
4. Changing leaders’ attitudes or beliefs about appropriate leadership and management styles and behaviours, through the enculturation inherent in *Lean* leadership training;
5. Demanding increased levels of expertise, accountability and commitment from leaders;
6. Measuring and using data effectively to identify actual and relevant local problems and the root causes of problems, rather than making assumptions;
7. Creating or supporting a ‘learning organization’ culture in which mistakes are opportunities for learning; consistent implementation of no-blame approaches to mistakes and errors.

The following discussion describes the evidence on which each of the hypotheses were based and draws primarily from the interviews. Quotes from the interviews are italicized. Participants are identified first by letter, denoting health region, and then by number, denoting order of interviews.

### Aligning the aims and objectives of health regions across the province

Hoshin Kanri is a strategic planning system that was designed to be consistent with *Lean*. It has been implemented alongside *Lean* in Saskatchewan. While a detailed program theory diagram has not been developed for Hoshin Kanri to date, our overarching program theory identifies it as the key strategy which is intended to generate a single, coherent, system-wide strategic plan. Using Normalization Process Theory [19] as an analytic framework, Hoshin Kanri might act as a component of context that enables, constrains, resources and realizes people and procedures with the goal of changing people’s actions. Concurrent implementation of *Lean* and Hoshin Kanri should, in theory, enable leaders across the system to work towards the goals established through the Hoshin Kanri process. There was some evidence in the interviews to suggest just how this might happen. There was widespread agreement among participants that efforts to improve health care quality were badly needed. “*The health care system is broken, and it’s got to be fixed, so there’s a lot at stake*” (B-01). Given that each health region prior to implementation of *Lean* set their own priorities and had their own distinct culture, policies and procedures, the “Hoshin Kanri” process of strategic planning for the province as a whole has the potential to create a seismic shift. *“That’s a huge change in health care…to make a whole system in a whole province work as one… the strategic deployment that now tries to pull together the whole province and be working on similar things” *(A-07)*.*

The fact that leaders from all regions underwent the same training program *“levelled the playing field”* (A-02) between individuals with differing experience and backgrounds, including physicians, and was valued by most participants. *“We’re all being exposed to the same philosophy, the same management, the same ideas…We’re talking the same language”* (B-01). *“Learning how to do things the same… will be the success”* (A-01) of the *Lean* initiative. *“We have managers who are great clinicians [but] really don’t have the basic fundamental competencies or management skills. Lean is making or enabling these managers to think differently than they have in the past”* (D-06).

New relationships between leaders with common interests but from different regions emerged as a result of the province-wide adoption of *Lean “There have been a lot more provincial meetings…One of the beneficial things…it has done is allowed me to meet a lot of other people in the province that work in similar areas. It’s helped with networking and actually having discussions around some of the system-wide issues”* (C-01). A disadvantage, though, was the need to attend meetings in person, which added the burden of significant travel for health regions located outside major urban centres.

Particularly for smaller and less well-resourced health regions, however, the new unified approach also engendered significant drawbacks. Before *Lean*, the smaller regions *“were their own leaders…they could choose what they wanted to do, how in depth they wanted to do it…that where a lot more of the satisfaction came from”* (D-01). Neither did the smaller health regions necessarily have the resources available to support *Lean* implementation: this is discussed further below. Some respondents in smaller health region also felt somewhat adrift in relation to the manner in which priorities and directions were being established. “*I’m not sure who out there, somewhere, is determining what we’re focusing on”* (A-03).

Implications for Future Research: We postulate that, in regions where leaders verbalized loss of control, change in focus and reduced focus on local issues of concern created by centralization, there is potential to lead to alienation and possibly disengagement from this quality improvement initiative, resulting in less support and less alignment between Hoshin Kanri and regional *Lean* initiatives. Evaluation of this element of program theory may assist in determining how a common set of strategic goals affect priorities at the regional and health unit level, and the linkages between those priorities, leadership engagement in Lean, leadership practices, and outcomes for staff and patients. This in turn suggests that the common goals intended through Hoshin Kanri would be more difficult to achieve.

### Authorizing attention and resources to quality improvement and change management

Rather than prioritizing day-to-day management, leaders were given the legitimate authority under *Lean* to spend their time on quality improvement initiatives*. “Part of the problems and frustrations that I think we’ve had for years is, the leadership is essentially putting out a lot of fires and there’s not time to actually do quality improvement work”* (C-01).

Leaders with a significant investment in *Lean* also identified a change of focus as necessary – moving from focusing on discrete targets or projects, to a sustained effort to change organizational culture. *Lean* was definitely not seen as a “quick fix” for entrenched systemic problems. *“In order for this model to really sink in to all of us, cause we’ve spent lots of money, we really need to put a lot a time and energy into it – help it grow and build the culture”* (C-01). Incremental change was seen as key to successful implementation of *Lean*. When asked about the outcomes of *Lean* activities, a manager indicated that they”*definitely had some gains, some not as significant as others, but every gain, is in the right direction…we are building on the successes”* (A-07).

Aside from the local process improvements that may have resulted from *Lean* activities and tools, leaders were now taking on responsibility for the “trickle down” cultural changes that gradually occurred with engaging more stakeholders in *Lean* activities. “*Projects are what really makes [Lean] live and breathe and makes it real for people…otherwise it feels like something that is done to them. This isn’t a new flavor of the day. This is a new world”* (B-04)*.*

The support leaders provided went beyond discrete tasks to recognize the struggles staff may be experiencing with the changes in process and culture brought about by *Lean. “Their [staff’s] world has changed. The processes have changed… how things are done has changed, which is absolutely challenging”* (A-03)*.* Leaders often needed to deal with concerns about *Lean* implementation from their staff, who expressed concerns to leaders about the investment of time, energy and money into *Lean. “If they could… do one thing different, it would be not pushing Lean so hard on the staff…. Because the [staff] said it just becomes overwhelming, and…they just can’t do it”* (D-01). Leaders took on the role of educating and reassuring staff that *Lean* was not a passing fad. *“The staff don’t wanna be part of something that might just go out the window in two years…part of the challenge with implementing Lean is the perception that Lean may not be a sustainable kind of thing”* (C-05)*.*

Variability in staff acceptance of and engagement in *Lean* implementation also required leaders to support the adoption of new attitudes, which could prove burdensome. *“Negative people, it’s just they don’t get it…unfortunately some of my staff went into it thinking, ‘How’s this going to affect my work? How this’s going to affect my job?’ But that’s not what it’s all about.”* (A-04). Particularly challenging were staff who were resistant to change. A front-line clinician noted the role of leaders in dealing with staff who disagreed about the value of *Lean*: *“There is still kind of a group that’s kind of pro-Lean and on that bandwagon and there’s a group that’s a bit more resistant …there’s always some smoothing out to be done between the two… change is always hard.” (*D-04). New skills in change management and staff support were needed to lead implementation of *Lean. “Not so much Lean, but it’s introducing massive change…some people handle it more easily that others…on the positive side, we’re all talking the same language, we’re taught the same things” *(B-01).

Some leaders found it rewarding to witness their staff beginning to make use of *Lean* principles and tools. We suggest one of the key mechanisms that can underpin *Lean* implementation is the development of key opinion leaders within the ranks of staff. “*Those [staff] that have learned to see are saying ‘I’m not going back’. They kind of pull the rest forward… I think when you’re in that mode, even though the workloads are heavy, you’re excited about your work or what you can accomplish…The bus has left the station. We’re not going back”* (A-07). Encouraging the participation and reinforcing the behaviors of staff who were supportive of *Lean* was another strategy used by leaders.

Implications for Future Research: In program theory terms, we posit that authorizing attention and resources to be spent on quality improvement activities will increase the time and attention that leaders do spend on quality improvement. This in turn will ensure that staff participate in such activities and that over time, a culture of continuous improvement is built. As the commitment is managers’ time and resources is significant, the extent to which day-to-day operations is seen to benefit or suffer as a result of this shift in managerial focus can be expected to affect the degree to which quality improvement activities will be sustainable. Furthermore, the level of managerial autonomy may also affect the extent of this shift; those with more autonomy may be less likely to persist with prioritizing quality improvement activities if the perceived benefits do not outweigh the perceived disadvantages. A realist evaluation of this element of the theory may identify whether increased leadership attention to quality improvement varies across levels of leadership and whether and in what circumstances this focus is maintained. It will also identify other contextual factors that are necessary in order for leaders to sustain a focus on building a culture of quality improvement.

### An integrated set of tools

*Lean* was valued by most participants for providing leaders with a set of structured, ‘common sense’ processes and tools to derive quantitative evidence and conduct key measurements of quality related to locally relevant issues. Inherent in the *Lean* program is an integrated set of tools for process improvement, which attempts to create structure for and instill confidence in leaders to tackle change management and legitimized leadership attention on quality-related activities. *“Lean pulls it altogether…we weren’t at that phase of…it all being connected together”* (A-07). Leaders who were early on in their *Lean* enculturation process, however, expressed a lack of self-confidence even after some training. *“You’re often feeling like you’re unsure. I don’t like as a leader feeling unsure… I just want to know what I need to do. I don’t like not knowing…when you don’t know, it’s really hard to feel good about yourself and the work you’re doing” (A-03).*

Most of the leaders we interviewed valued the structure imposed by adoption of *Lean*. “*We all know that Japanese culture traditionally has brought dedication, commitment and discipline…and we need that ‘cause we’re too loosey-goosey”* (B-01). This sentiment was not, however, shared by all participants. At least one found the specific processes involved with *Lean* implementation as carried out in the province rigid and prescriptive: *“very military…it was built as a very military model type thing, but that was a little over the edge”* (C-06)*.* The focus of some *Lean* activities on timing certain procedures was not always welcomed and seemed misguided for participants who were less *Lean*-enculturated: *“Well, maybe we should be taking care of all of the big garbage first, before we worry about the seconds… I really disagree on timing tasks and things like that, I really disagree on that*.” (D-01).

Gaining expertise in *Lean* activities also provided opportunities for those not in formal leadership roles to gain some recognition as an informal leader. *“[I’m a] little fish in the big pond, but I think maybe it’s been kind of a way to gain recognition and respect …just kind of making myself known and recognized a little bit has been a nice, a nice advantage.”* (D-02)

Implications for Future Research: Lean program theory proposes that use of a common set of *Lean* tools will ensure consistent quality improvement processes and practices across the province, which will in turn generate distributed quality improvement knowledge, thereby generating improved decision-making ultimately leading to better quality of care. It further proposes that providing leaders with training in the use of all *Lean* tools will allow them not only to choose the right tool for the job, but also to use multiple tools simultaneously to generate quality improvement synergies. Experience in use of the tools will improve skills in inclusive leadership, while experiences of success will build belief in and commitment to the *Lean* approach, contributing to a virtuous cycle. A realist evaluation will investigate to which, and the circumstances in which, this program theory plays out in practice. Leaders who value the structure Lean provides are more likely to trial a wider range of tools. As a result of trialing more tools, the leaders are more likely to achieve success in meeting their objectives and therefore more likely to establish the virtuous cycle. Leaders with high levels of autonomy are more likely to choose tools that they believe are appropriate to local issues. In circumstances where there is agreement between different levels of leadership about the more important issues to address, the most appropriate tools are likely to be selected, successes are most likely to be achieved, again establishing a virtuous cycle. When there is disagreement between different levels of leadership, however, the imposition of “resisted tools” will build further resistance and antagonism, instead setting up a vicious cycle.

### Changing attitudes or beliefs about appropriate leadership and management styles

*Lean* espouses particular roles and management styles for leaders. *Lean* leaders are intended to operate foremost as coaches and mentors, rather than as administrators or directors, thereby increasing their teams’ expertise both directly in their work and in quality improvement itself. They are intended to develop inclusive approaches that both inform and seek input from all members of teams, thus creating a culture in which it is safe for any staff member to speak up about issues or offer ideas. They are expected to increase the time that they spend ‘on the floor’, thereby improving their understanding of issues on the floor and improving their relationships with staff, both of which should contribute to quality improvement. They are also expected to adopt and ensure ‘no blame’ approaches in response to mistakes and errors, making it possible for people to speak up about things that have gone wrong so that they can be prevented in future; and they are supposed to lead by example, both modelling what is expected and encouraging like behavior by staff.

The importance of having strongly supportive leadership in *Lean* implementation was clearly articulated by participants. One director noted that “*Without the leaders being supportive and helping us to get things done, it [Lean implementation] won’t succeed”* (A-01)*.* Leaders “model the way” for staff and have the ability to set quality improvement agendas at the local level. They also muster the human and material resources (to the extent of their authority) required to support the implementation of *Lean* initiatives.

In discussing the unsuccessful introduction of *Lean* in a previous workplace, a staff member noted that there had been very little information provided to staff by management. *“Looking back, I wonder…whether management didn’t buy into it”* (B-05). From our range of interviews, the “buy-in” by, or enculturation of, leaders appeared to reflect a dose–response relationship to some extent, whereby the most senior leaders, who had undergone more training than the junior leaders, seemed to have the greatest comfort with and were the biggest proponents of *Lean*.

Leaders’ roles were perceived by some participants to be evolving towards the coaching-focused orientation characteristic of a learning organization. A director indicated he now asked *“What kinds of things can I do to assist the [staff] and support them?…You’re removing the barriers.”* A staff member in other site commented on greater inclusivity, suggesting that her relationship with her manager had been “*definitely strengthened*”, because the manager now allowed greater say in decision-making for the staff:

*“…we, as the staff, get to make some…decisions, … put us more in control of our department, … we were out there, we were making changes, we were deciding what we wanted, what we didn’t want” (D-09).*

These changed practices by leadership are a critical aspect of the program theory for *Lean*, not least because they contribute to empowerment of staff to play their intended roles.

The renewed emphasis on “customers’ needs and desires” in *Lean* also meant that leaders needed to take serious account of the way in which patients were being affected by health care practices. *Lean* “*inherently embeds a more patient-centred approach, because it’s all about patient flow*” (B-01). One leader reported that having patients involved ‘*changes the direction of what we thought we were gonna do’* and by increasing awareness of the whole patient experience, reinforced the importance of the patient experience:

*“…We see it through their eyes, coming through a process …you know, you always just think it’s…your appointment, not recognizing all of the work flow that has to happen, to make that appointment occur, and then the follow up for you to get the care you need.”* (A-07)

This same leader also identified that involving patient representatives in *Lean* change processes provided ‘insight’ into the way patients interpreted services, including health promotion materials on the walls:

*“Even though we know some of those messages do work to … alert people …but when you’re stressed and, and anxious, being made to… see those kinds of messages is not really the first thing you should see as you, come to a place for care. So you know, I think we’re all learning those little pieces that make a difference to the patients.” (A-07)*

Part of the leadership change involved increased visibility of leaders on the “shop floor”. *“We have more emphasis on being…at the place where the work takes place…we can’t lead from behind. You need to have [leaders] that are visible”* (B-01). Visibility of leaders can signify to workers that the work done on the “shop floor” is of such high value that leaders prioritize these visits over the many other demands on their time. Leaders being present at the work site also conveys that they are willing to witness and to participate in the day-to-day realities and demands to which workers are subjected, which in turn can help leaders account for these realities in their own decision-making. *“We do see our leaders more, which is a good thing, because all of a sudden, instead of just a name, it’s a name and a face and somebody who is working right side by side with the staff, so that’s been the change so that’s been the good with the leaders” (D-01).*

However, some leaders reported major discrepancies between implementation processes they experienced and the espoused values of *Lean*. They reported lack of clear information even when it was specifically requested, lack of ability to set local priorities, and lack of flexibility to adapt tools to local contexts.

Implications for Future Research: We hypothesize that leaders are less likely to adopt prescribed new leadership styles or they may be more likely to revert to previous styles of leadership if they perceive either of the following: a) the lack of centralized support to support the prescribed management style: or b) the failure of central authorities to accept situations where the leadership style may need to be modified to reflect local contexts. If the central authority is unwilling to accommodate suggestions from the leader regarding most suitable management practices, this may cause the leader to question the central authority’s understanding of the local context and erode the leader’s confidence in the *Lean* leadership style.

### Demanding increased levels of expertise, accountability and commitment from leaders

Leaders commonly demonstrated a high level of commitment to ensuring the success of *Lean* implementation and felt accountable to do their best, particularly with regards to removing barriers to quality improvement initiatives. The personal contribution of leaders was recognized as critically important to success of *Lean*, but also more broadly to the salvation of health care in the province. The focus on the individual accountability of each was intensified as *Lean* was implemented. *“Every person going through the certification realizes the accountability they hold when they start one of these workshops or one of these processes”* (A-01).

This accountability extended to ensuring that those in leadership positions were in fact committed to its implementation. One senior leader explained the need to ensure the team’s commitment to the *Lean* management system: *“We’ve had some managers leave…that weren’t the best fit for us progressing… If you’ve got someone who’s gonna be an impediment to improvement or a structure that’s an impediment to improvement, the onus is on myself as a leader to create some degree of change”*(C-05). The need for cohesion amongst leaders was echoed by A-04: *“If you’re not ready for [change], then this might not be the right place for you to be working”*.

There were significant pressures, from within leaders themselves but also from the system (including the government as funders), throughout the *Lean* implementation process – *“we can’t afford to fail”* (B-01). Intense training is provided through *Lean* which is intended to ensure that leaders have the skills they need for the role and a deep understanding of how the various components of *Lean* fit together to generate change. However, the very intensity of that training was problematic for some. As one respondent noted, “*It was very sad to see people almost fainting because they were so scared they wouldn’t do well…I saw co-workers that I felt so bad for that were crying, that were hyperventilating, that were sick”* (C-06)*.* In spite of the intense training, some managers still felt unprepared to lead *Lean* activities. “*We were shooting in the dark [on the manager’s first Rapid Process Improvement Workshop]. There were 2 nights where I couldn’t sleep…Usually I’m very easy at punching out. But the RPIW Team Lead position…it was probably the hardest thing*” (A-04).

While leaders had an increased level of accountability, the resources needed to do the work were not always available, particularly in smaller health regions. *“We have thrown a bit of money here and there…you realize that you absolutely need more resources”* (A-03). Supports such as IT resources to pull required data and team level supports to help run the *Lean* projects were not uniformly available and created additional pressure on leaders. *“More quality improvement data, collection data analysis support, to the leaders of the teams, I think that if we would have had some more of that earlier on, that might of not made the pace feel so frantic”* (A-07). The increased accountability but lack of resources meant that “*They [leaders, staff and patients] spend all this time, they come up with …all these great ideas, and then, sometimes it just seems to kind of like… put on the shelf … and the changes that are made aren’t sustained…. We’ve gotten our hands slapped a little bit from [the consultants] …because…we spent all this time doing Mistake Proofing projects, but there was no follow up”* (D-06).

Several leaders remarked on the lack of role clarity and ambiguous nature of accountability for some activities. “*We just sort of struggle with what’s the big picture because … we get to one spot and then we’ll move to another spot…you’re often feeling like you’re unsure. … I don’t want to question the process, in that I believe in it…but I just want to know what I need to do. …When you don’t know, it’s really hard to feel good about yourself and the work you’re doing”*.

Implications for Future Research: Increased levels of staff and patient participation in improvement activities and increased visibility of leaders all contribute to increasing transparency, which in turn acts to hold leaders accountable for improvement. Our program theory suggests that leaders are more likely to implement *Lean* effectively, and adopt the desired management styles, when they themselves are empowered, having the appropriate autonomy, information, support, access to resources and access to professional development.

We hypothesize that leaders are less likely to adopt the desired leadership styles when they experience dissonance between the espoused values and principles of *Lean* and its implementation (such as a lack of information from higher levels of leadership about priorities and directions and a lack of autonomy) and/or where there is poor ‘vertical integration’ between the priorities at central, regional and site levels, generating perceived lack of relevance at the local level.

### Measuring and using data effectively

*Lean* management is predicated on high visibility and better use of better data. It requires leaders to collect and display locally relevant data using techniques such as data walls, and to design quality improvements to address the problems identified through their data. Use of the appropriate *Lean* tools then means that the causes of safety problems are addressed, which contributes to improved patient safety. Data also works to hold leaders accountable for safety and quality improvements.

Measurement was valued by front line, physician and leader respondents. A front line staff member said *“I’m a big proponent of measuring things…not making change unless you know there’s a reason for the change…I’m quite keen on improvements made though measurement”.* The data generated through *Lean* activities was a tool seen by some leaders as a means to promote fairness in decision-making. “*It’s not so much who can squeak louder. I think it goes more really where the evidence is and where the actual needs need to be”* (C-06).

Leaders also saw the value of measurement in providing irrefutable evidence about why change was necessary to staff and to assure themselves that change was necessary. “*When people challenge you on the data… Not sort of going with a sort of hunch about what we believe is wrong, but actually doing some time observations and data collection…so that we are very sure we know what the problem is”* (A-03) Measurement also acted to correct perceptions about the exact nature of problems and to provide evidence of the success of changes, thus providing a positive feedback loop to reinforce engagement.

Physicians also appreciated, and were reported by others to appreciate, the objective nature of the evidence provided by *Lean* activities. One of the doctors indicated that *“Physicians like to see data around things… evidence around things…[Lean] lends itself very well to physician groups”.* This is important because physician engagement was described as necessary for specific changes to be implemented and valued. The importance of physician engagement was highlighted in the Conference Board of Canada survey as a key success factor [3, 30]. Engaged physicians encouraged buy-in by others and modelled new behaviors, particularly for other doctors.

Implications for Future Research: We hypothesize that leaders will be more supportive of *Lean* initiatives in a context of timely access to meaningful data relevant to their projects. Where data is difficult to access, unavailable, perceived as poor quality or challenging to obtain or collect, leader support of Lean will be compromised.

### Creating or supporting the development of a ‘learning organization’ culture

*Lean* seeks to create an environment in which mistakes are opportunities for learning, with consistent implementation of no-blame approaches to mistakes and errors. The *Lean* philosophy embeds the concept of “fail forward fast”, stressing that virtually nothing succeeds fully the first time and rapid experimentation allows for quick identification of failures and problem-solving to get back on track [31]. Some interviews acknowledged this intent: *“Even if we make mistakes, even if we realize we have used a tool that’s maybe the wrong tool, that’s a learning for us”* (C-05). *“So if it doesn’t work, you find out in a month that it doesn’t work, you don’t have to keep it”* (A-01).

Argyris and Schön [33] posit that learning involves correction and detection of error [27]. Argyris (1978; 1993) suggests that double-loop learning is essential if practitioners and organizations are to make informed decisions in ambiguous and rapidly changing contexts [27, 32]. Single-loop learning occurs when people search for another strategy to address the error and work with, or within, the governing variables, so that extant goals, values, plans and rule are operationalized rather than questioned. *Single-loop learning* typically follows pre-set routines and plans, engendering less risk for both the person and the organization and affording greater control.

Some of the *Lean* tools, which provide highly structured ways of addressing particular problems in very specific contexts, may be considered to support single loop learning. Similarly, ‘standard work’ products from those tools may be considered a single loop learning outcome in that they provide a highly structured way of undertaking particular work. In contrast, *double-loop learning* leads to questioning of the governing variables themselves, such that governing variables may be altered and a shift in strategies and consequences occurs. The underlying philosophy of *Lean* and the changes it envisages to leadership style, staff empowerment and patient-centered design of services and systems involve learning of this kind. This is more challenging for many people and might be expected to take longer to evolve.

Implications for Future Research: We hypothesize that leaders will support Lean to the extent that believe that they are able to make mistakes without negative consequences for their positions within the organization.

## Other findings from the baseline interviews

In addition to the materials on which our program theory is based, the baseline interviews revealed perspectives on a number of other matters which are likely to affect whether, where and how *Lean* works.

## Attitudes towards *Lean*

*Lean* as a quality improvement tool was often favorably compared with previous quality improvement efforts*. “Lean is probably the best tool that the ministry’s implemented in all of my years. It makes sense”* (C-06). Past experience with quality improvement initiatives, however, made some leaders wary of investing too heavily in *Lean. “I hope it doesn’t get dropped… If it is a long-term commitment, then we should be learning from history”* (D-01).

However, another participant suggested that the messaging around *Lean* was a problem: *“If they [government and senior leaders] would message it as, this is about …improving quality, I totally, get that. But when they start talking about this is about reducing costs, and then you’ve got all these people, who are sitting there saying, we’re not reducing costs, it kind of takes away from the credibility of the whole Lean initiative”* (D-06).

Changing organizational culture depends on changing attitudes and norms, and action-opinion theories, such as Festinger’s cognitive dissonance theory (1957), suggest that attitude change can follow from (rather than precede) behavior change [28]. Festinger’s (1957) theory is based on three main assumptions: a) humans are sensitive to inconsistencies between actions and beliefs; b) recognition of the inconsistency creates dissonance, which motivates the person to resolve the dissonance; and c) dissonance can be resolved in one of three ways (changing beliefs; changing actions; changing perceptions of actions) [28]. Changes in behavior that are mandated (in this case, by workplace expectations that employees participate in *Lean* activities) may lead to situations in which an individual may resolve dissonance by developing a more positive appraisal of *Lean* than previously held. Over time and with many individuals being exposed to *Lean* activities, this strategy may foster organizational change.

Both reservations – the concern about sustained implementation and that about messaging – reflect the importance of credibility of the program to ensure sustained leadership engagement in *Lean* implementation.

## Intensity of the implementation process

The ‘frantic’ pace of *Lean* implementation was seen as both a blessing and a curse for leaders. A number of participants who were managers reported the need to live in “two different worlds” as *Lean* was being implemented – carrying on with day-to-day administrative duties they remained accountable for as well as moving forward with the implementation of *Lean. “I’d be lying if it [biggest challenge] wasn’t the workload…and the ongoing workload…We have three big things…our daily job, which was full-time before…now the Lean leader training…and everyone’ doing projects that are involving you in one way or another…There’s shrapnel flying around all the time” *(A-04). *“It’s hard to keep up the pace…it is that fire under your feet because we cannot stop…Having said that, the other thing is if you don’t get the momentum, I’m not sure we would be getting same gains” *(A-07)*.* Several respondents had spent vacation time engaged in *Lean* activities because no other time was available. Others reported a significant amount of personal time spent working after hours because of the demands of *Lean* implementation. The high demands placed upon leaders can reasonably be expected to result in changes to their level of engagement and may affect sustainability of this initiative, particularly in the long term.

The positive side of rapid implementation was that those who have been trained could quickly implement their skills in *Lean* projects and see results in a short amount of time. The negative side is that some leaders reported being *“overloaded, overwhelmed and overworked”* (B-03) and recognize that their staff are experiencing change fatigue. *“It’s hard to keep up. It is hard for our staff”* (A-03). Leaders who recognize that the changes brought about by *Lean* have a negative impact on staff workload and morale may be required to invest additional time and resources in supporting those individuals. This would be important to nurture and sustain the efforts of staff to achieving the goals of *Lean*.

## Short-term outcomes from *Lean* activities

Despite the early stage of implementation at the time these interviews were conducted, a number of interviews identified short-term outcomes from the use of *Lean* tools. *“All of them [Lean projects] are further ahead than they would’ve been before we started. So, even if it’s not the perfect results we were looking for, or the best bang for the buck…it is still better than what we had”* (A-01).

## Conclusions

While the implementation of *Lean* in Saskatchewan is at an early stage, this study has generated initial hypotheses and realist program theory that can form the basis for future evaluation of *Lean* initiatives. Theoretical work on learning organizations that encompasses the notion of single and double loop learning [27, 32] has been valuable in understanding some key components of *Lean* implementation, although this work may also present challenges, both to the *Lean* implementation strategy adopted in Saskatchewan or to the program theory we have developed to represent it. As discussed above, *Lean* appears to involve elements of both single loop and double loop learning encompassing – perhaps – some perceived contradictions. On the one hand, *Lean* uses highly structured tools and generates highly structured standard work in the local setting (single loop learning). On the other, *Lean* aims to build a culture that is data-based, questioning and reflective, and incorporates a responsibility to challenge peers and authority figures (double loop learning). The *Lean* implementation process in Saskatchewan similarly incorporates particular tensions: it represents a top-down attempt to build bottom up processes of improvement. This, in turn, creates inherent tensions for leaders. On the one hand, they are required to implement *Lean*: its implementation is non-negotiable despite any dissatisfaction that it (or the lack of additional resourcing for implementation) may generate for staff. On the other, they are encouraged to empower staff (albeit only in relation to particular aspects of practice) and to develop more open and responsive leadership processes. These tensions are not, in fact, irreconcilable, but they require a degree of sophistication and skill to manage, as well as the establishment of particular cultural mores.

Our current overarching program theory reflects the ‘train leaders first’ implementation approach used in Saskatchewan, suggesting that developing leadership capacity and culture will precede other systemic changes. Perhaps, however, there will need to be significant changes in both leadership and staff behaviors before such attitudinal and cultural change can be observed. If this is the case, it is likely that change evolves through multiple small feedback loops (a small behavior change generated through use of a particular *Lean* tool generates a small attitudinal change amongst participants, which provides a more enabling context for a next behavior change which enables some further attitudinal change, and so on). If this is the case, it has significant implications for the order in which particular indicators of change should be expected and therefore the bases on which progress towards effectiveness should be judged. It also has significant implications for the processes of planning and implementation that should be encouraged to improve patient outcomes in the longer term.

This paper has dealt specifically with the changes in leadership associated with implementation of the Saskatchewan model of Lean. Much further work remains to be accomplished in examining the impact of implementation on other key groups, such as front-line workers, patients and families. Future challenges involve examining the way Lean is or is not embedded and integrated into health care in Saskatchewan.
